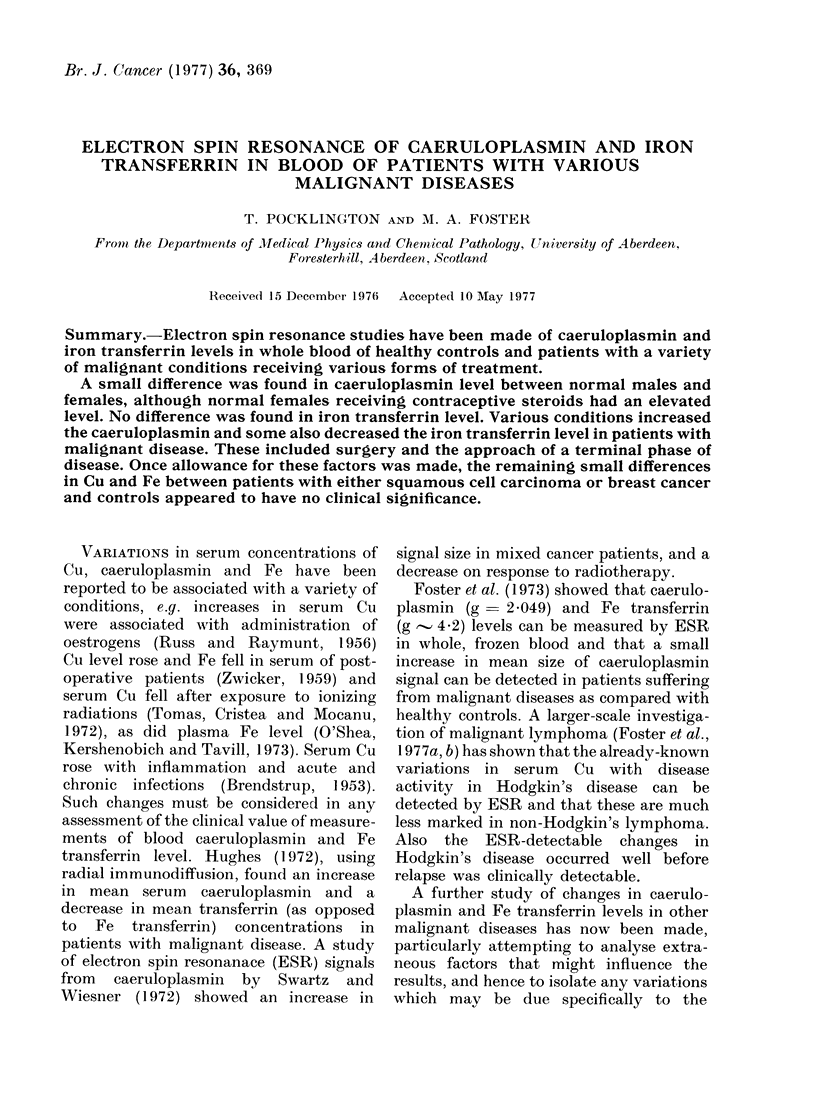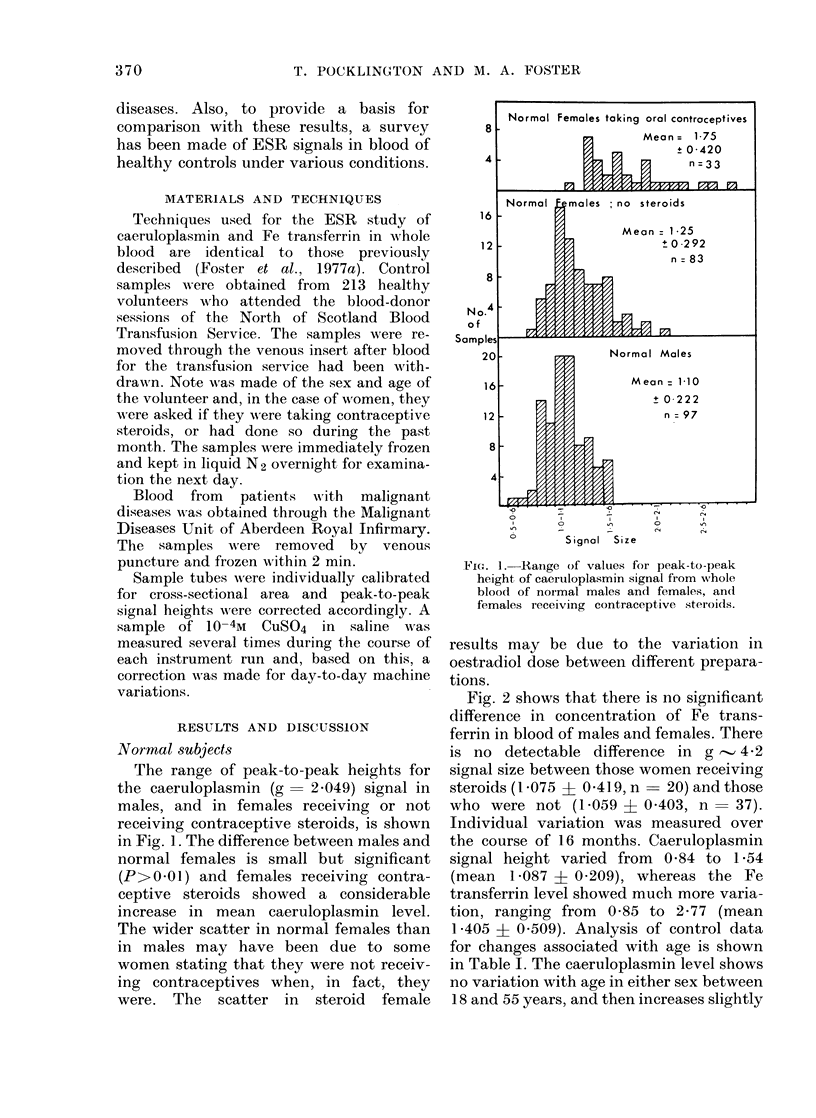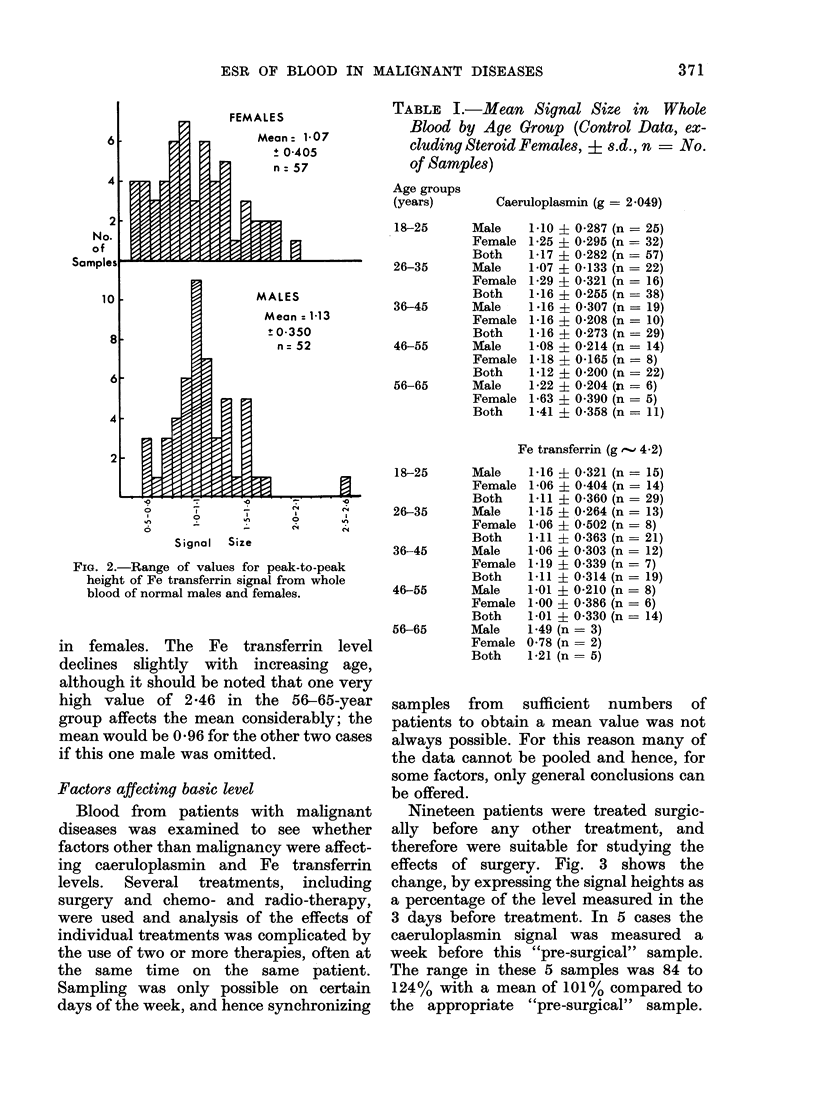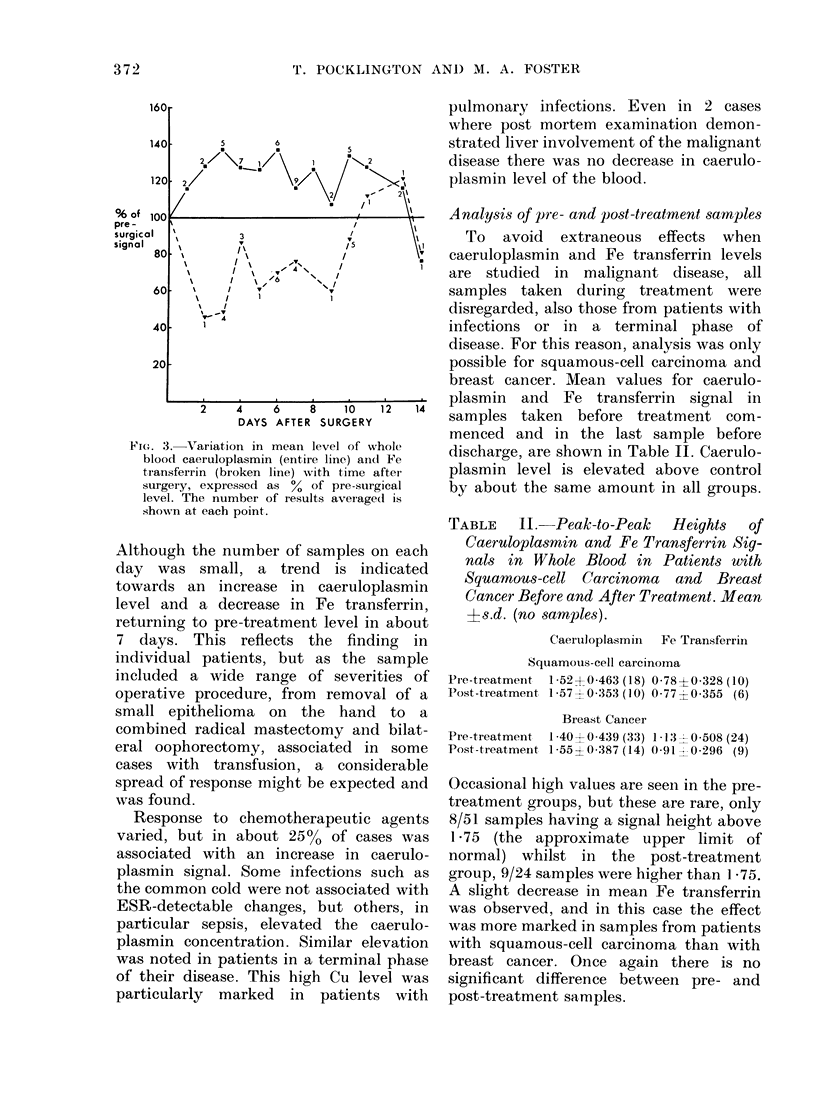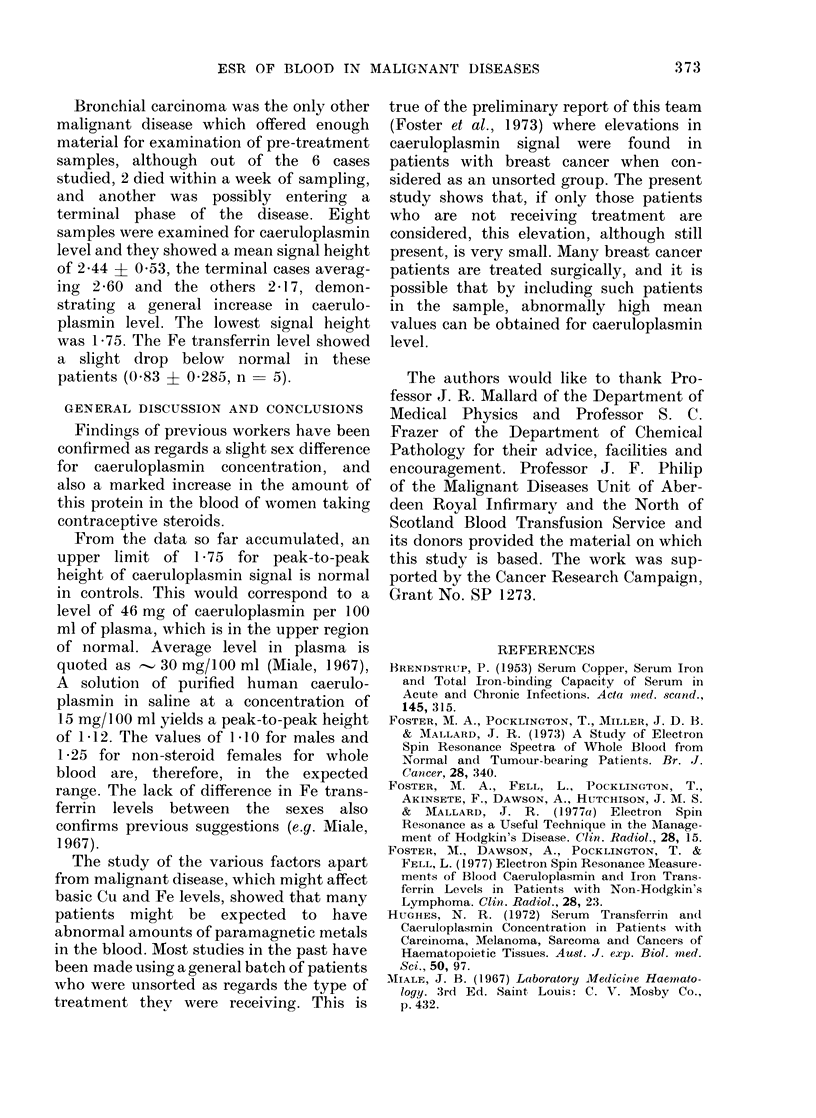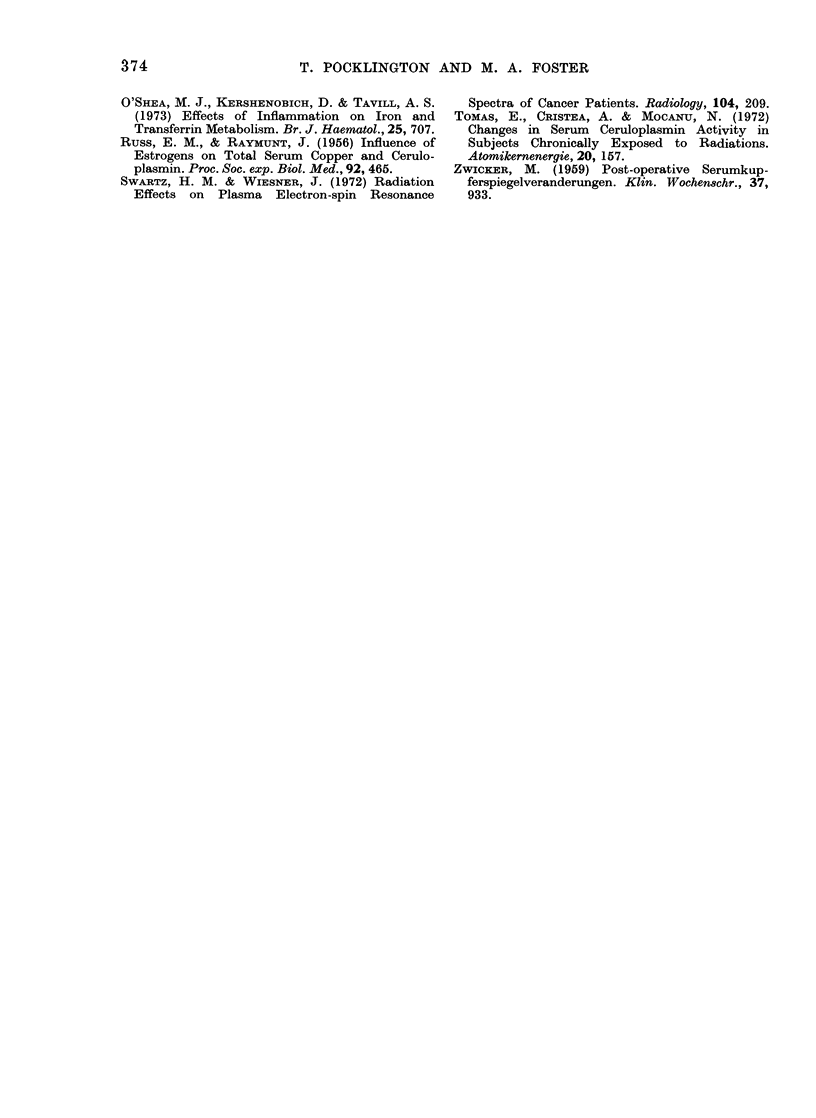# Electron spin resonance of caeruloplasmin and iron transferrin in blood of patients with various malignant diseases.

**DOI:** 10.1038/bjc.1977.202

**Published:** 1977-09

**Authors:** T. Pocklington, M. A. Foster

## Abstract

Electron spin resonance studies have been made of caeruloplasmin and iron transferrin levels in whole blood of healthy controls and patients with a variety of malignant conditions receiving various forms of treatment. A small difference was found in caeruloplasmin level between normal males and females, although normal females receiving contraceptive steroids had an elevated level. No difference was found in iron transferrin level. Various conditions increased the caeruloplasmin and some also decreased the iron transferrin level in patients with malignant disease. These included surgery and the approach of a terminal phase of disease. Once allowance for these factors was made, the remaining small differences in Cu and Fe between patients with either squamous cell carcinoma or breast cancer and controls appeared to have no clinical significance.


Br. J. Cancer (1977) 36, 369

### ELECTRON SPIN RESONANCE OF CAERULOPLASMIN AND IRON TRANSFERRIN IN BLOOD OF PATIENTS WITH VARIOUS MALIGNANT DISEASES

T. POCKLINGTON AND M. A. FOSTER

*From the Departments of Medical Physics and Chemical Pathology, University of Aberdeen, Foresterhill, Aberdeen, Scotland*

Received 15 December 1976  Accepted 10 May 1977

**Summary.—Electron spin resonance studies have been made of caeruloplasmin and iron transferrin levels in whole blood of healthy controls and patients with a variety of malignant conditions receiving various forms of treatment.**

**A small difference was found in caeruloplasmin level between normal males and females, although normal females receiving contraceptive steroids had an elevated level. No difference was found in iron transferrin level. Various conditions increased the caeruloplasmin and some also decreased the iron transferrin level in patients with malignant disease. These included surgery and the approach of a terminal phase of disease. Once allowance for these factors was made, the remaining small differences in Cu and Fe between patients with either squamous cell carcinoma or breast cancer and controls appeared to have no clinical significance.**

VARIATIONS in serum concentrations of Cu, caeruloplasmin and Fe have been reported to be associated with a variety of conditions, *e.g.* increases in serum Cu were associated with administration of oestrogens (Russ and Raymunt, 1956) Cu level rose and Fe fell in serum of post-operative patients (Zwicker, 1959) and serum Cu fell after exposure to ionizing radiations (Tomas, Cristea and Mocanu, 1972), as did plasma Fe level (O'Shea, Kershenobich and Tavill, 1973). Serum Cu rose with inflammation and acute and chronic infections (Brendstrup, 1953). Such changes must be considered in any assessment of the clinical value of measurements of blood caeruloplasmin and Fe transferrin level. Hughes (1972), using radial immunodiffusion, found an increase in mean serum caeruloplasmin and a decrease in mean transferrin (as opposed to Fe transferrin) concentrations in patients with malignant disease. A study of electron spin resonanace (ESR) signals from caeruloplasmin by Swartz and Wiesner (1972) showed an increase in signal size in mixed cancer patients, and a decrease on response to radiotherapy.

Foster *et al.* (1973) showed that caeruloplasmin ($g = 2{\cdot}049$) and Fe transferrin ($g \sim 4{\cdot}2$) levels can be measured by ESR in whole, frozen blood and that a small increase in mean size of caeruloplasmin signal can be detected in patients suffering from malignant diseases as compared with healthy controls. A larger-scale investigation of malignant lymphoma (Foster *et al.*, 1977*a*, *b*) has shown that the already-known variations in serum Cu with disease activity in Hodgkin's disease can be detected by ESR and that these are much less marked in non-Hodgkin's lymphoma. Also the ESR-detectable changes in Hodgkin's disease occurred well before relapse was clinically detectable.

A further study of changes in caeruloplasmin and Fe transferrin levels in other malignant diseases has now been made, particularly attempting to analyse extraneous factors that might influence the results, and hence to isolate any variations which may be due specifically to the diseases. Also, to provide a basis for comparison with these results, a survey has been made of ESR signals in blood of healthy controls under various conditions.

### MATERIALS AND TECHNIQUES

Techniques used for the ESR study of caeruloplasmin and Fe transferrin in whole blood are identical to those previously described (Foster *et al.*, 1977*a*). Control samples were obtained from 213 healthy volunteers who attended the blood-donor sessions of the North of Scotland Blood Transfusion Service. The samples were removed through the venous insert after blood for the transfusion service had been withdrawn. Note was made of the sex and age of the volunteer and, in the case of women, they were asked if they were taking contraceptive steroids, or had done so during the past month. The samples were immediately frozen and kept in liquid $N_2$ overnight for examination the next day.

Blood from patients with malignant diseases was obtained through the Malignant Diseases Unit of Aberdeen Royal Infirmary. The samples were removed by venous puncture and frozen within 2 min.

Sample tubes were individually calibrated for cross-sectional area and peak-to-peak signal heights were corrected accordingly. A sample of $10^{-4}$M $CuSO_4$ in saline was measured several times during the course of each instrument run and, based on this, a correction was made for day-to-day machine variations.

### RESULTS AND DISCUSSION

### *Normal subjects*

The range of peak-to-peak heights for the caeruloplasmin ($g = 2{\cdot}049$) signal in males, and in females receiving or not receiving contraceptive steroids, is shown in Fig. 1. The difference between males and normal females is small but significant ($P > 0{\cdot}01$) and females receiving contraceptive steroids showed a considerable increase in mean caeruloplasmin level. The wider scatter in normal females than in males may have been due to some women stating that they were not receiving contraceptives when, in fact, they were. The scatter in steroid female

FIG. 1.—Range of values for peak-to-peak height of caeruloplasmin signal from whole blood of normal males and females, and females receiving contraceptive steroids.

results may be due to the variation in oestradiol dose between different preparations.

Fig. 2 shows that there is no significant difference in concentration of Fe transferrin in blood of males and females. There is no detectable difference in $g \sim 4{\cdot}2$ signal size between those women receiving steroids ($1{\cdot}075 \pm 0{\cdot}419$, $n = 20$) and those who were not ($1{\cdot}059 \pm 0{\cdot}403$, $n = 37$). Individual variation was measured over the course of 16 months. Caeruloplasmin signal height varied from 0·84 to 1·54 (mean $1{\cdot}087 \pm 0{\cdot}209$), whereas the Fe transferrin level showed much more variation, ranging from 0·85 to 2·77 (mean $1{\cdot}405 \pm 0{\cdot}509$). Analysis of control data for changes associated with age is shown in Table I. The caeruloplasmin level shows no variation with age in either sex between 18 and 55 years, and then increases slightly

FIG. 2.—Range of values for peak-to-peak height of Fe transferrin signal from whole blood of normal males and females.

TABLE I.—*Mean Signal Size in Whole Blood by Age Group (Control Data, excluding Steroid Females, ± s.d., n = No. of Samples)*

| Age groups (years) | | Caeruloplasmin (g = 2·049) |
|---|---|---|
| 18–25 | Male | 1·10 ± 0·287 (n = 25) |
| | Female | 1·25 ± 0·295 (n = 32) |
| | Both | 1·17 ± 0·282 (n = 57) |
| 26–35 | Male | 1·07 ± 0·133 (n = 22) |
| | Female | 1·29 ± 0·321 (n = 16) |
| | Both | 1·16 ± 0·255 (n = 38) |
| 36–45 | Male | 1·16 ± 0·307 (n = 19) |
| | Female | 1·16 ± 0·208 (n = 10) |
| | Both | 1·16 ± 0·273 (n = 29) |
| 46–55 | Male | 1·08 ± 0·214 (n = 14) |
| | Female | 1·18 ± 0·165 (n = 8) |
| | Both | 1·12 ± 0·200 (n = 22) |
| 56–65 | Male | 1·22 ± 0·204 (n = 6) |
| | Female | 1·63 ± 0·390 (n = 5) |
| | Both | 1·41 ± 0·358 (n = 11) |
| | | Fe transferrin (g ~ 4·2) |
| 18–25 | Male | 1·16 ± 0·321 (n = 15) |
| | Female | 1·06 ± 0·404 (n = 14) |
| | Both | 1·11 ± 0·360 (n = 29) |
| 26–35 | Male | 1·15 ± 0·264 (n = 13) |
| | Female | 1·06 ± 0·502 (n = 8) |
| | Both | 1·11 ± 0·363 (n = 21) |
| 36–45 | Male | 1·06 ± 0·303 (n = 12) |
| | Female | 1·19 ± 0·339 (n = 7) |
| | Both | 1·11 ± 0·314 (n = 19) |
| 46–55 | Male | 1·01 ± 0·210 (n = 8) |
| | Female | 1·00 ± 0·386 (n = 6) |
| | Both | 1·01 ± 0·330 (n = 14) |
| 56–65 | Male | 1·49 (n = 3) |
| | Female | 0·78 (n = 2) |
| | Both | 1·21 (n = 5) |

in females. The Fe transferrin level declines slightly with increasing age, although it should be noted that one very high value of 2·46 in the 56–65-year group affects the mean considerably; the mean would be 0·96 for the other two cases if this one male was omitted.

*Factors affecting basic level*

Blood from patients with malignant diseases was examined to see whether factors other than malignancy were affecting caeruloplasmin and Fe transferrin levels. Several treatments, including surgery and chemo- and radio-therapy, were used and analysis of the effects of individual treatments was complicated by the use of two or more therapies, often at the same time on the same patient. Sampling was only possible on certain days of the week, and hence synchronizing samples from sufficient numbers of patients to obtain a mean value was not always possible. For this reason many of the data cannot be pooled and hence, for some factors, only general conclusions can be offered.

Nineteen patients were treated surgically before any other treatment, and therefore were suitable for studying the effects of surgery. Fig. 3 shows the change, by expressing the signal heights as a percentage of the level measured in the 3 days before treatment. In 5 cases the caeruloplasmin signal was measured a week before this "pre-surgical" sample. The range in these 5 samples was 84 to 124% with a mean of 101% compared to the appropriate "pre-surgical" sample.

FIG. 3.—Variation in mean level of whole blood caeruloplasmin (entire line) and Fe transferrin (broken line) with time after surgery, expressed as % of pre-surgical level. The number of results averaged is shown at each point.

Although the number of samples on each day was small, a trend is indicated towards an increase in caeruloplasmin level and a decrease in Fe transferrin, returning to pre-treatment level in about 7 days. This reflects the finding in individual patients, but as the sample included a wide range of severities of operative procedure, from removal of a small epithelioma on the hand to a combined radical mastectomy and bilateral oophorectomy, associated in some cases with transfusion, a considerable spread of response might be expected and was found.

Response to chemotherapeutic agents varied, but in about 25% of cases was associated with an increase in caeruloplasmin signal. Some infections such as the common cold were not associated with ESR-detectable changes, but others, in particular sepsis, elevated the caeruloplasmin concentration. Similar elevation was noted in patients in a terminal phase of their disease. This high Cu level was particularly marked in patients with pulmonary infections. Even in 2 cases where post mortem examination demonstrated liver involvement of the malignant disease there was no decrease in caeruloplasmin level of the blood.

*Analysis of pre- and post-treatment samples*

To avoid extraneous effects when caeruloplasmin and Fe transferrin levels are studied in malignant disease, all samples taken during treatment were disregarded, also those from patients with infections or in a terminal phase of disease. For this reason, analysis was only possible for squamous-cell carcinoma and breast cancer. Mean values for caeruloplasmin and Fe transferrin signal in samples taken before treatment commenced and in the last sample before discharge, are shown in Table II. Caeruloplasmin level is elevated above control by about the same amount in all groups.

TABLE II.—*Peak-to-Peak Heights of Caeruloplasmin and Fe Transferrin Signals in Whole Blood in Patients with Squamous-cell Carcinoma and Breast Cancer Before and After Treatment. Mean* $\pm$*s.d. (no samples).*

| | Caeruloplasmin | Fe Transferrin |
|---|---|---|
| Squamous-cell carcinoma | | |
| Pre-treatment | 1·52±0·463 (18) | 0·78±0·328 (10) |
| Post-treatment | 1·57±0·353 (10) | 0·77±0·355 (6) |
| Breast Cancer | | |
| Pre-treatment | 1·40±0·439 (33) | 1·13±0·508 (24) |
| Post-treatment | 1·55±0·387 (14) | 0·91±0·296 (9) |

Occasional high values are seen in the pre-treatment groups, but these are rare, only 8/51 samples having a signal height above 1·75 (the approximate upper limit of normal) whilst in the post-treatment group, 9/24 samples were higher than 1·75. A slight decrease in mean Fe transferrin was observed, and in this case the effect was more marked in samples from patients with squamous-cell carcinoma than with breast cancer. Once again there is no significant difference between pre- and post-treatment samples.

Bronchial carcinoma was the only other malignant disease which offered enough material for examination of pre-treatment samples, although out of the 6 cases studied, 2 died within a week of sampling, and another was possibly entering a terminal phase of the disease. Eight samples were examined for caeruloplasmin level and they showed a mean signal height of $2 \cdot 44 \pm 0 \cdot 53$, the terminal cases averaging 2·60 and the others 2·17, demonstrating a general increase in caeruloplasmin level. The lowest signal height was 1·75. The Fe transferrin level showed a slight drop below normal in these patients ($0 \cdot 83 \pm 0 \cdot 285$, $n = 5$).

### GENERAL DISCUSSION AND CONCLUSIONS

Findings of previous workers have been confirmed as regards a slight sex difference for caeruloplasmin concentration, and also a marked increase in the amount of this protein in the blood of women taking contraceptive steroids.

From the data so far accumulated, an upper limit of 1·75 for peak-to-peak height of caeruloplasmin signal is normal in controls. This would correspond to a level of 46 mg of caeruloplasmin per 100 ml of plasma, which is in the upper region of normal. Average level in plasma is quoted as $\sim$ 30 mg/100 ml (Miale, 1967), A solution of purified human caeruloplasmin in saline at a concentration of 15 mg/100 ml yields a peak-to-peak height of 1·12. The values of 1·10 for males and 1·25 for non-steroid females for whole blood are, therefore, in the expected range. The lack of difference in Fe transferrin levels between the sexes also confirms previous suggestions (*e.g.* Miale, 1967).

The study of the various factors apart from malignant disease, which might affect basic Cu and Fe levels, showed that many patients might be expected to have abnormal amounts of paramagnetic metals in the blood. Most studies in the past have been made using a general batch of patients who were unsorted as regards the type of treatment they were receiving. This is true of the preliminary report of this team (Foster *et al.*, 1973) where elevations in caeruloplasmin signal were found in patients with breast cancer when considered as an unsorted group. The present study shows that, if only those patients who are not receiving treatment are considered, this elevation, although still present, is very small. Many breast cancer patients are treated surgically, and it is possible that by including such patients in the sample, abnormally high mean values can be obtained for caeruloplasmin level.

The authors would like to thank Professor J. R. Mallard of the Department of Medical Physics and Professor S. C. Frazer of the Department of Chemical Pathology for their advice, facilities and encouragement. Professor J. F. Philip of the Malignant Diseases Unit of Aberdeen Royal Infirmary and the North of Scotland Blood Transfusion Service and its donors provided the material on which this study is based. The work was supported by the Cancer Research Campaign, Grant No. SP 1273.

### REFERENCES

BRENDSTRUP, P. (1953) Serum Copper, Serum Iron and Total Iron-binding Capacity of Serum in Acute and Chronic Infections. *Acta med. scand.*, **145,** 315.

FOSTER, M. A., POCKLINGTON, T., MILLER, J. D. B. & MALLARD, J. R. (1973) A Study of Electron Spin Resonance Spectra of Whole Blood from Normal and Tumour-bearing Patients. *Br. J. Cancer*, **28,** 340.

FOSTER, M. A., FELL, L., POCKLINGTON, T., AKINSETE, F., DAWSON, A., HUTCHISON, J. M. S. & MALLARD, J. R. (1977*a*) Electron Spin Resonance as a Useful Technique in the Management of Hodgkin's Disease. *Clin. Radiol.*, **28,** 15.

FOSTER, M., DAWSON, A., POCKLINGTON, T. & FELL, L. (1977) Electron Spin Resonance Measurements of Blood Caeruloplasmin and Iron Transferrin Levels in Patients with Non-Hodgkin's Lymphoma. *Clin. Radiol.*, **28,** 23.

HUGHES, N. R. (1972) Serum Transferrin and Caeruloplasmin Concentration in Patients with Carcinoma, Melanoma, Sarcoma and Cancers of Haematopoietic Tissues. *Aust. J. exp. Biol. med. Sci.*, **50,** 97.

MIALE, J. B. (1967) *Laboratory Medicine Haematology.* 3rd Ed. Saint Louis: C. V. Mosby Co., p. 432.

O'Shea, M. J., Kershenobich, D. & Tavill, A. S. (1973) Effects of Inflammation on Iron and Transferrin Metabolism. *Br. J. Haematol.*, **25**, 707.
Russ, E. M., & Raymunt, J. (1956) Influence of Estrogens on Total Serum Copper and Ceruloplasmin. *Proc. Soc. exp. Biol. Med.*, **92**, 465.
Swartz, H. M. & Wiesner, J. (1972) Radiation Effects on Plasma Electron-spin Resonance Spectra of Cancer Patients. *Radiology*, **104**, 209.
Tomas, E., Cristea, A. & Mocanu, N. (1972) Changes in Serum Ceruloplasmin Activity in Subjects Chronically Exposed to Radiations. *Atomikernenergie*, **20**, 157.
Zwicker, M. (1959) Post-operative Serumkupferspiegelveranderungen. *Klin. Wochenschr.*, **37**, 933.